# Vascular mechanisms underlying the hypotensive effect of *Rumex acetosa*

**DOI:** 10.1080/13880209.2018.1446031

**Published:** 2018-03-21

**Authors:** Hafiz Misbah-Ud-Din Qamar, Rahila Qayyum, Umme Salma, Shamim Khan, Taous Khan, Abdul Jabbar Shah

**Affiliations:** Department of Pharmacy, Cardiovascular Research Group, COMSATS Institute of Information Technology, Abbottabad, Pakistan

**Keywords:** Calcium channel blocker, NO-mediated vasorelaxant, antihypertensive

## Abstract

**Context:***Rumex acetosa* L. (Polygonaceae) is well known in traditional medicine for its therapeutic efficacy as an antihypertensive.

**Objective:** The study investigates antihypertensive potential of crude methanol extract (Ra.Cr) and fractions of *Rumex acetosa* in normotensive and hypertensive rat models and probes the underlying vascular mechanisms.

**Materials and methods:** Ra.Cr and its fractions were tested *in vivo* on normotensive and hypertensive Sprague-Dawley rats under anaesthesia for blood pressure lowering effect. *In vitro* experiments on rat and *Oryctolagus cuniculus* rabbit aortae were employed to probe the underlying vasorelaxant mechanism.

**Results:** In normotensive rats under anaesthesia, Ra.Cr caused fall in MAP (40 mmHg) at 50 mg/kg with % fall of 27.88 ± 4.55. Among the fractions tested, aqueous fraction was more potent at the dose of 50 mg/kg with % fall of 45.63 ± 2.84. In hypertensive rats under similar conditions, extract and fractions showed antihypertensive effect at same doses while aqueous fraction being more potent, exhibited 68.53 ± 4.45% fall in MAP (70 mmHg). In isolated rat aortic rings precontracted with phenylephrine (PE), Ra.Cr and fractions induced endothelium-dependent vasorelaxation, which was partially blocked in presence of l-NAME, indomethacin and atropine. In isolated rabbit aortic rings pre-contracted with PE and K^+^-(80 mM), Ra.Cr induced vasorelaxation and shifted Ca^2+^ concentration–response curves to the right and suppressed PE peak formation, similar to verapamil, in Ca^2+^-free medium.

**Discussion and conclusions:** The data indicate that l-NAME and atropine-sensitive endothelial-derived NO and COX enzyme inhibitors and Ca^2+^ entry blocking-mediated vasodilator effect of the extract explain its antihypertensive potential.

## Introduction

Since ancient times, *Rumex* species (dock) have been well known for their use in traditional medicine having therapeutic efficacy and countless medicinal importance (Babulka 2004). *Rumex acetosa* L. (Polygonaceae) is a perennial herb, commonly known as Sorrel or Common sorrel (Lee et al. 2005). It has global distribution in Europe, North America, Africa and Asia (Pakistan), particularly in the northern areas (Vasas et al. 2015). The plant has been included officially in the Korean Food Code (Korea Food & Drug Administration) as one of the important food items used in folk medicine (Lee et al. 2005). Juice made from the leaves of *R. acetosa* is used to reduce high blood pressure (BP) (Qureshi et al. 2007). As a food, its leaves are utilized in sauces and salads (Alfawaz 2006). Its therapeutic usefulness has also been documented in a variety of conditions, such as hypertension and diseases of respiratory, skin, gastric and nervous systems (Song and Hu 1999; Duke 2002; Vasas et al. 2015). *R. acetosa* has some pharmacological activities, including anti-inflammatory, antioxidant (Wegiera et al. 2007), antitumor, antibacterial, antiviral and antifungal properties (Taylor et al. 1996; Demirezer et al. 2001; Lee et al. 2005). However, Sun et al. (2015) found that the antihypertensive effect of *R. acetosa* ethanol extract is mediated through NO production. This investigation has neither worked out fractionation of the crude extract into pharmacologically active fractions nor identified other mechanisms in the parent crude extract. Recently, Ahmad et al. (2016) reported anticholinesterase activity of essential oil from *R. hastatus*. We assumed that *R. acetosa* might possess such constituents and/or activities and act as a cholinomimetic. Substances that increase acetylcholine activity (Heymans 1960) are known to decrease BP and vascular resistance. We studied in detail the effect of *R. acetosa* methanol extract on BP in salt-induced hypertensive and normotensive rats in the absence and presence of atropine. Further studies in isolated vascular preparations were carried out to explore additional vascular mechanisms of the extract and fractions.

## Materials and methods

### Plant materials

Fresh leaves of *Rumex acetosa* (RA) were collected during flowering season in September 2013 from the forests in District Swat, Khyber Pakhtunkhwa (KPK), Pakistan and authenticated by Mr. Mehboob Ur Rehman, Associate Professor, Department of Botany, PG Jehanzeb College, Saidu Sharif, Swat, Pakistan. A voucher specimen was retained under the code Ra-L-09/13 in the Graduate Research Lab of Pharmacology and Pharmacognosy, Department of Pharmacy, COMSATS Institute of Information Technology (CIIT), Abbottabad, Pakistan vide Notification EC/PHM/07-2013/CIIT/ATD.

### Preparation of the crude extract from the leaves of *Rumex acetosa* and fractionation

Shade dried leaves (4 kg) were homogenized into powder with herbal grinder and macerated in 7.0 L methanol (70%) for three days with occasional shaking in amber glass container. The material was passed through a muslin cloth and finally filtered through a Whatman #1 filter paper. The procedure was repeated thrice and the combined filtrate was evaporated in rotary evaporator coupled with chiller and water bath under reduced pressure at 35–40 °C. A semisolid mass of *Rumex acetosa* crude extract (Ra.Cr) was obtained with yield of 7.8% w/w (312 g).

Furthermore, activity-directed fractionation of the methanol crude extract was carried out to see shift in activity from the parent crude extract to any of the fractions. As described previously (Williamson et al. 1998), a known quantity of the extract (150 g) was mixed up with 400 mL distilled water to make it flowable. This was then poured out into a separating funnel and equal volume of *n-*hexane was added. The mixture was shaken vigorously, regularly allowing the air to escape out. It was kept for about 30–45 min to let the two layers separate. The upper layer of *n*-hexane was acquired and the same procedure was repeated twice and all the *n*-hexane layers were collected and concentrated in a rotary evaporator to obtain the *n*-hexane fraction (Ra.nHex). The procedure was repeated with other organic solvents, such as chloroform and ethyl acetate. Final remnant layer was regarded as aqueous layer. All layers obtained were concentrated on rotary evaporator. Yield (w/w) of *n*-hexane (Ra.nHex), chloroform (Ra.Chlor), ethyl acetate (Ra.EtAc) and aqueous (Ra.Aq) fractions was 24.8% (37.3 g), 15.7% (23.55 g), 9.6% (14.4 g) and 48.5% (72.75 g), respectively. Approximately, 1.4% extract lost in the process. The crude extract and fractions were stored in air tight glass containers at –4 °C. Crude extract and fractions were dissolved in appropriate solvents and dilutions were made fresh, for use in the *in vivo* and *in vitro* studies, on the day of experiment.

### Drugs and standards

The following drugs and standards were procured from the source specified: acetylcholine chloride ≥98% (Alfa Aesar Gmbh & Co, Karlsruhe, Germany), atropine sulphate monohydrate >98% (Fluka-AG, Buchs, Switzerland), potassium chloride, phenylephrine (PE) hydrochloride ≥98%, norepinephrine bitartrate ≥98%, Nω-nitro-l-arginine methyl ester (l-NAME) hydrochloride ≥98%, verapamil hydrochloride ≥99%, indomethacin hydrochloride (Sigma-Aldrich Inc., St. Louis, MO) and ethylene glycol-O-O′-bis(2-aminoethyl)-N,N,N′,N′-tetraacetic acid (EGTA) ≥ 97% (Alfa Aesar, Heysham, UK). Stock solutions of all the chemicals were made in distilled water and diluted freshly when required.

### Experimental animals

All the experiments performed complied with the rulings of Institute of Laboratory Animal Resources, Commission on Life Sciences (National Research Council 1996) and approved by the Ethical Committee, CIIT, Abbottabad, Pakistan. Sprague-Dawley (SD) rats (200–250 g, *n* = 60) and local rabbits (1–1.5 kg, *n* = 6), not standardized, used in the study were bred and housed in the animal house of Department of Pharmacy, CIIT, Abbottabad under a controlled environment (23–25 °C). Animals were provided with standard food and water *ad libitum*.

### *In vivo* blood pressure measurement in anesthetized rats

#### Normotensive rats

As described (Gilani et al. 2005), the invasive BP measurement experiments were performed on male SD rats (200–250 g; *n* = 30). Rats were anesthetized with an intraperitoneal injection of thiopental sodium (50–90 mg/kg), minor midtracheal surgical incision (approximately 1 cm) was made to expose trachea, carotid artery and right jugular vein. The trachea was cannulated with a polyethylene tubing PE-20 to maintain spontaneous respiration. The right jugular vein was cannulated with a polyethylene tubing PE-50 to infuse standard drugs and test materials. The carotid artery was cannulated with similar tubing filled with heparinized saline (60 IU/mL) and connected to a pressure transducer (MLT 0699) coupled with bridge amplifier (N12128) and PowerLab (ML 846) Data Acquisition System (ADInstruments, Sydney, Australia). This connection was used for recording and measurement of mean arterial BP. The exposed surface for the cannulation was covered with a piece of tissue paper moistened in warm saline. Body temperature of the rat was maintained by using an overhead lamp.

#### Hypertensive rats

Protocols of Lawler et al. (1987) and Vasdev et al. (2003) were followed with some modifications. Sprague-Dawley male rats (200–250 g; *n* = 30) were hygienically housed in uniform conditions. The rats were given high-salt (8% NaCl) diet and water *ad libitum* for 8 weeks. One day prior to experiment, the rats were given normal diet and water. Subsequently, the rats were used for *in vivo* BP measurement. Rats having BP 150–190 mmHg were considered as hypertensive and employed for experimentations.

### Experimental protocols

The rats were stabilized for 20–30 min; acetylcholine and norepinephrine were used to assure the stability of the rats towards hypotensive and hypertensive responses, respectively. Acetylcholine (1 µg/kg) 0.1 mL was gradually injected followed by a flush of 0.1 mL normal saline, which caused a fall in mean arterial pressure (MAP). Approximately, 5–10 min later, when the normal pattern of BP was attained, norepinephrine (1 µg/kg) was slowly injected followed by a flush of 0.1 mL normal saline, which caused an increase in MAP. When the normal pattern of BP resumed, rats were injected intravenously with 0.1 mL normal saline or with the same volume of test substances. The MAP was allowed to return to the resting level between injections. Doses of drugs, extract and fractions were adjusted in 0.1 mL volume and injected intravenously followed by a flush of 0.1 mL normal saline. Changes in BP were calculated as a difference between the steady-state values prior and after injection. The MAP was calculated as the diastolic BP plus one third pulse width (systolic BP–diastolic BP). % fall in MAP was compared with the pretreated values and also compared different doses among themselves.

### Vascular reactivity studies in isolated aortic rings

#### Rat thoracic aorta

As described previously (Sánchez-Salgado et al. 2007; Shah and Gilani 2010), the thoracic aortic rings from SD rats were used to see, specifically, effect of the extract and fractions on vascular tone. SD rats were sacrificed by cervical dislocation. Thoracic aorta was isolated, cleaned of fat and connective tissue and made into rings of 2–3 mm width. Each individual ring was carefully hooked between two stainless steel probes and suspended in a 10 mL tissue bath containing normal Kreb’s solution warmed at 37 °C and aerated with carbogen (5% CO_2_ in O_2_). The composition of Kreb’s solution was (mM): NaCl 118.2, NaHCO_3_ 25.0, CaCl_2_ 2.5, KCl 4.7, KH_2_PO_4_ 1.3, MgSO_4_ 1.2 and glucose 11.7 (pH 7.4). A preload of 1 g was placed on each aortic ring. Changes in isometric tension were recorded and analysed through a force transducer (MLT 0201) coupled with a bridge amplifier (N12128) and PowerLab (ML 846) Data Acquisition System (ADInstruments, Sydney, Australia). Aortic rings were allowed to equilibrate for 30–45 min. In some aortic rings, endothelium was deliberately removed by rubbing the luminal surface with forceps and were considered denuded when acetylcholine exhibited relaxation <10%. Aortic rings were pre-contracted with PE (1 µM) and effect of cumulative addition of extract and fraction was determined.

### Effect of extract and fractions on rat aortic tone

To investigate the involvement of NO, prostacyclin and vascular muscarinic receptors, endothelium-intact rings were pre-incubated for 15–20 min with l-NAME (10 µM), indomethacin (1 µM) and atropine (1 µM). The effect of the extract and fractions was determined in the absence and presence of these inhibitors.

### Effect on K^+^ (80 mM)-induced contractions

To see possible effect on vascular smooth muscle tone and Ca^2+^ movements through voltage-gated calcium channels, aortic rings were pre-contracted with K^+^ (80 mM). Cumulative addition of the extract and fractions was made and % relaxation was calculated, as maximum of high K^+^ pre-contractions.

### Rabbit thoracic aorta

As described previously (Chan et al. 2006; Shah and Gilani 2009), these protocols were specifically used to see effect of the extract and fractions on Ca^2+^ movements through membrane and store-operated Ca^2+^ channels. Rabbits were killed by a blow on the back of head; the thoracic aorta was removed and cut into rings of approximately 2–3 mm width. The tissues were suspended in normal Kreb’s solution, maintained at 37 °C, and aerated continuously with carbogen. A basal tension of 2 g was placed on each aortic ring and equilibrated for 45–60 min. Phenylephrine (1 µM) was used to stabilize the preparations. Changes in isometric tension were recorded and analysed through a force transducer (MLT 0201) coupled with a bridge amplifier (N12128) and PowerLab (ML 846) Data Acquisition System (AD Instruments, Sydney, Australia).

### Effect of extract and fractions on rabbit aortic tone

The protocol of Chan et al. (2006) was followed with some modifications. Phenylephrine (1 µM) or K^+^ (80 mM) was used to induce steady-state contractions. The extract and fractions were added in a cumulative manner to obtain sigmoidal concentration response curves (CRCs) and the relaxation was expressed as % of induced contractions. These protocols allowed us to have an indirect approach to study effect of the extract and fractions on voltage-gated calcium channels or receptor-operated calcium channels (ROCs) and Ca^2+^ releases from cellular internal store(s).

### Calcium channel blocking activity

In a set of experiments, an attempt was made to assure if the relaxation induced by the extract and fractions involved Ca^2+^ influx through voltage-gated calcium channels. Aortic rings were washed four to five times with Ca^2+^-free medium before the control sigmoidal CRCs of CaCl_2_. After control sigmoidal CRCs of CaCl_2_ were reproduced, aortic rings were pretreated with the extract, fractions (0.01–10 mg/mL) and verapamil (1 µM) for 30–45 min to test the possible calcium channel blocking effect. A parallel vehicle control was also run under similar experimental conditions.

### Effect on intracellular Ca^2+^ stores and baseline tension

In a set of experiments, the aim was to clarify whether the relaxation induced by extract and its fractions is related to inhibition of intracellular Ca^2+^. The rings were exposed to Ca^2+^-free medium for 15 min before the application of PE (1 µM) to induce the first transient contraction. The composition of Ca^2+^-free medium was (mM): NaCl 118.2, NaHCO_3_ 25.0, KCl 4.7, KH_2_PO_4_ 1.3, MgSO_4_ 1.2, EGTA (0.05 mM) and glucose 11.7 (pH 7.4). The rings were then washed three times with normal Kreb’s solution and incubated for at least 40 min for refilling of the intracellular Ca^2+^ stores. Subsequently, the medium was rapidly replaced with Ca^2+^-free medium and the rings were incubated for another 15 min. The second contraction was then induced by PE (1 µM) in the presence of extract and fractions (mg/mL), which were added 30 min before the application of PE, both contractions were compared. To see possible stimulant (vasoconstrictor) effect, extract and fractions were also tested on base line tension in Ca^2+^ free medium.

### Statistics

All the data are expressed in mean ± standard error of the mean (SEM), and the median effective concentrations (EC_50_ values) are given with 95% confidence intervals (CI). The statistical parameter applied is Student’s *t*-test with *p* < 0.05 noted as significantly different. The significance of differences between means was assessed by one-way ANOVA followed by a *post hoc* Tukey’s test. The results of different treatments were then plotted and adjusted by nonlinear curve fitting using computer software GraphPad Prism (GraphPad Software, San Diego, CA).

## Results

### Effect on blood pressure in normotensive and hypertensive anesthetized rats

**Figure 1.**
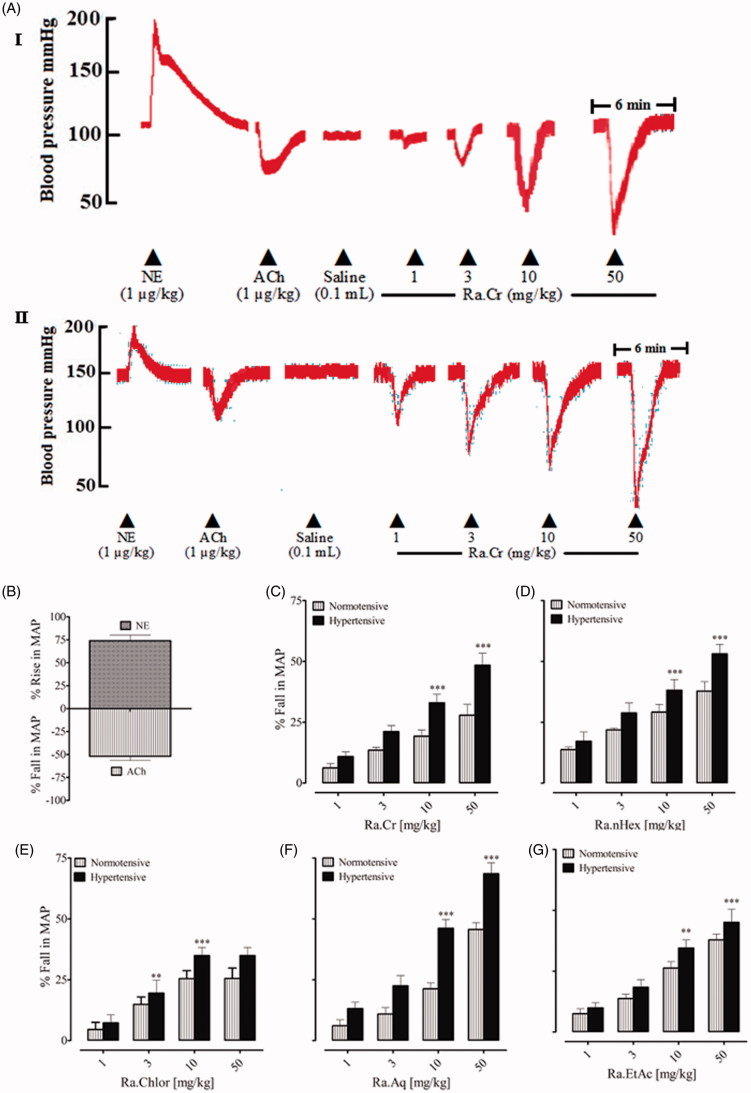
A representative tracing (A I) is showing the effect of norepinephrine (NE), acetylcholine (ACh) and the crude extract of *Rumex acetosa* (Ra.Cr) on blood pressure in normotensive and hypertensive rats (A II) under anaesthesia. (B) The hypertensive and hypotensive effects of NE and Ach, respectively. (C–G) The hypotensive response of the crude extract of Ra.Cr and its fractions in normotensive and hypertensive rats under anaesthesia. Values shown are mean ± SEM. Six determinations in either case. **p < 0.01, ***p < 0.001.

Before the injection of the crude extract and fractions of RA, the standard drugs such as acetylcholine and norepinephrine were used, they caused a fall and rise in MAP, respectively (Figure 1(B)). We found that MAP was 115 ± 5.12 (*n* = 20) and 166 ± 4.45 (*n* = 20) in normotensive and high salt-induced hypertensive rats, respectively.

In normotensive rats under anaesthesia, intravenous injection of Ra.Cr caused a fall in MAP (Figure 1(A)), at doses of 1, 3, 10 and 50 mg/kg with fall of 6.23 ± 1.78, 13.52 ± 1.15, 19.21 ± 2.58 and 27.88 ± 4.55%, respectively (Figure 1(C)). Among the fractions tested, all fractions caused a fall in MAP (Figure 1), aqueous fraction exhibited potent vasorelaxation at 50 mg/kg dose (45.63 ± 2.84) (Figure 1(G)). The % fall in each case was statistically different (*p* < 0.05) compared to pretreated values and among the different doses.

In hypertensive rats under anaesthesia, intravenous injection of the extract and fractions induced antihypertensive effect at same doses (Figure 1(A I)). However, the effect was statistically significant at doses of 10 and 50 mg/kg, in comparison to the normotensive rats. % fall in MAP observed at 1, 3, 10 and 50 mg/kg of Ra.Cr was 10.91 ± 1.89, 21.17 ± 2.42, 32.97 ± 3.51 and 48.40 ± 4.93 (Figure 1(C)). Among the fractions tested, all fractions caused fall in MAP (Figure 1). The aqueous fraction was found more antihypertensive at doses of 10 and 50 mg/kg, with % fall of 46.13 ± 3.57 and 68.53 ± 4.45 (Figure 1). The % fall in each case was statistically different (*p* < 0.05) at doses of 10 and 50 mg/kg in comparison to lower doses.

To see the possible effect of the extract and fractions on muscarinic receptors, rats were pretreated with atropine (1 mg/kg). In the atropinized rats, the BP lowering effect of the extract and fractions was abolished at lower doses while partially ablated at higher doses. The effect was statistically significant at doses of 10 and 50 mg/kg, in comparison to the normotensive atropinized rats. % fall in MAP observed at 1, 3, 10 and 50 mg/kg of Ra.Cr was 1.47 ± 0.38, 1.83 ± 0.76, 4.83 ± 2.36 and 9.16 ± 2.91. Among the fractions tested, all fractions caused fall in MAP at higher doses. The aqueous fraction was found more potent at doses of 10 and 50 mg/kg, with % fall of 11.68 ± 3.51 and 25.34 ± 5.13. The % fall in each case was statistically different (*p* < 0.05) at doses of 10 and 50 mg/kg in comparison to lower doses.

### Endothelium-dependent and-independent effects

**Figure 2.**
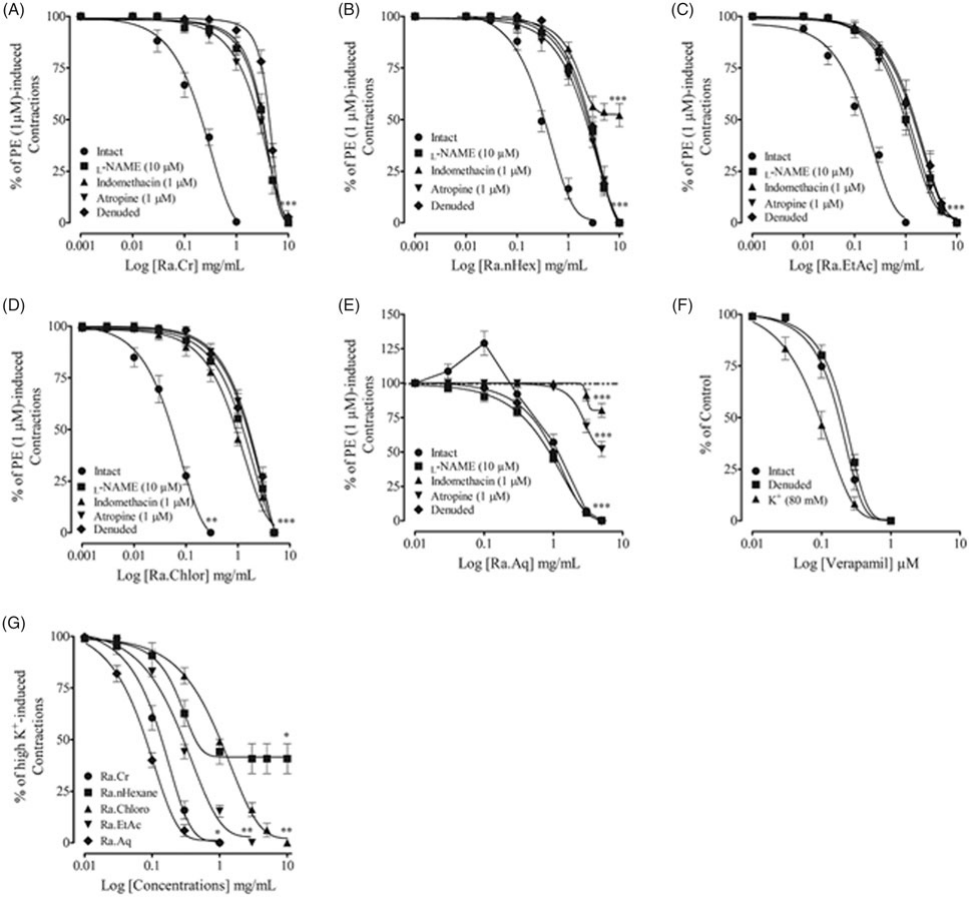
Response of the crude extract of *Rumex acetosa* (Ra.Cr) and its fractions on assorted parameters in isolated rat aorta preparations. (A–D) Endothelium-dependent vasorelaxant response of the crude extracts of *Rumex acetosa* (Ra.Cr); *n*-hexane (Ra.nHex); ethyl acetate (Ra.EtAc) and chloroform (Ra.Chlor) fractions in aortic rings precontracted with PE (1 µM). (E) Endothelium-dependent contractile and endothelium-independent relaxant effect of the aqueous (Ra.Aq) fraction in aortic rings precontracted with PE (1 mM). (F) The endothelium-independent vasodilator effect of verapamil. (G) The combined vasodilator effect of Ra.Cr, Ra.nHeaxane, Ra.Chlor, Ra.EtAc and Ra.Aq on K^+^ (80 mM)-induced contractions in isolated rat aorta rings. Values shown are mean ± SEM. Five to six determinations.

In rat aorta rings with intact endothelium pre-contracted with PE (1 µM), cumulative addition of Ra.Cr caused partial endothelium-dependent vasorelaxation with EC_50_ value of 0.32 mg/mL (0.21–0.42). Pre-treatment of intact aortic rings with l-NAME (10 µM), indomethacin (1 µM) and atropine (1 µM), partially inhibited vasorelaxation induced by Ra.Cr with overlapping EC_50_ value of 3.14 mg/mL (2.12–5.32). Moreover, in aorta rings with denuded endothelium pre-contracted with PE (1 µM), Ra.Cr induced relaxation at higher concentrations with EC_50_ value of 4.22 mg/mL (3.2–5.42) (Figure 2(A)).

Intact rat aorta rings precontracted with PE (1 µM), cumulative addition of Ra.nHex induced partial endothelium-dependent vasorelaxation. When endothelium intact aortic rings, pretreated with l-NAME (10 µM) and atropine, the relaxation was delayed and abolished by pretreatment with indomethacin (1 µM) (Figure 2(B)). Ethyl acetate and chloroform fractions also induced partial endothelium-dependent vasorelaxation, similar to the parent crude extract (Figure 2(C,D)). Interestingly, the effect of aqueous fraction on rat aortic tone was different. It caused endothelium-dependent vasoconstrictor effect at lower concentrations followed by vasorelaxation at higher concentrations (Figure 2(E)). In the denuded aortic rings, the vasoconstrictor effect was abolished. Aortic rings with intact endothelium, pretreated with l-NAME (10 µM), did not affect relaxation to the aqueous fraction and same was observed with denuded rings. However, intact aortic rings pretreated with indomethacin and atropine abolished relaxation to the aqueous fraction (Figure 2(E)).

Rat aortic rings precontracted with K^+^ (80 mM), cumulative addition of extract and fractions inhibited K^+^ (80 mM)-induced pre-contractions with varying potencies, aqueous fraction being more potent while *n*-hexane was least, which induced partial inhibition (Figure 2(G)). Verapamil, a typical calcium channel blocker induced endothelium-independent vasodilator effect with more potency against high K^+^ precontractions (Figure 2(F)).

### Effect on calcium channels and intracellular calcium stores

**Figure 3.**
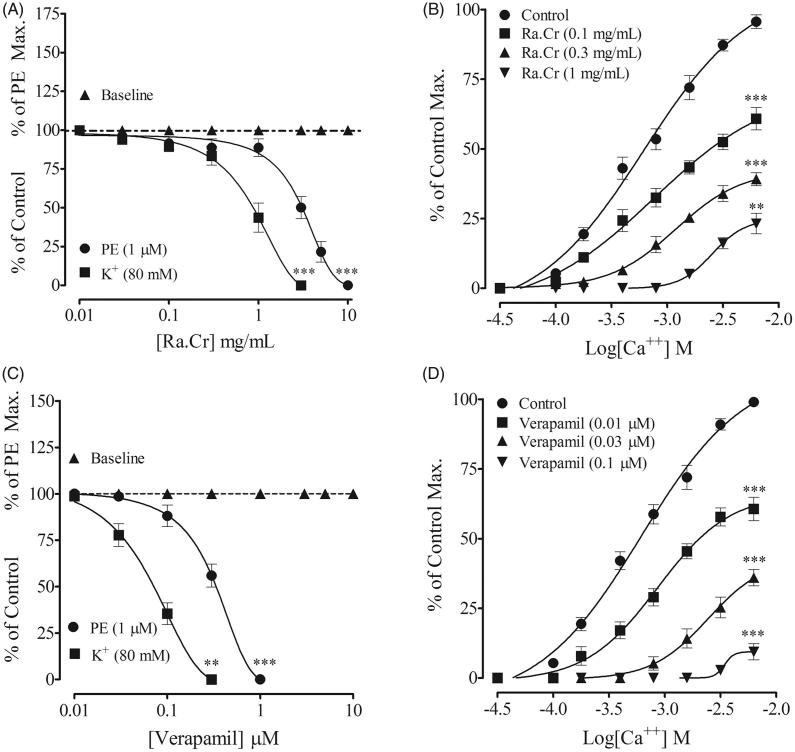
Concentration-dependent vasorelaxant response of (A) the crude extract of *Rumex acetosa* (Ra.Cr); (C) verapamil on phenylephrine (PE 1 µM) and K^+^ (80 mM) precontractions and on baseline tension, (B,D) respectively, their effect on the sigmoidal CaCl_2_ concentration–response curves, constructed in Ca^2+^-free medium, in isolated rabbit aorta preparations. Values shown are mean ± SEM. Five to six determinations.

Reponses of PE (1 µM) and K^+^ (80 mM) are more pronounced and reproducible in rabbit aortic rings. Rabbit aortic rings were used to see effect of the extract and fractions on Ca^2+^ movements through voltage-gated calcium channels and store-operated Ca^2+^ channels. Rabbit aortic rings pre-contracted with PE and high K^+^, extract was added cumulatively, which induced a vasodilator effect with more potency against high K^+^ (0.87 mg/mL (0.74–1.43) than PE (3.21 mg/mL (1.11–5.23) and, respectively, similar to verapamil (Figure 3(A,C)). Pre-incubation of the aortic rings with Ra.Cr (0.1–1.0 mg/mL) shifted the CaCl_2_ sigmoidal CRCs to the right (Figure 3(B)), constructed in Ca^2+^-free medium, similar to that caused by verapamil (Figure 3(D)). Ra.Cr and verapamil did not produce contractile effect when tested on basal line (Figure 3(A,C)).

**Figure 4.**
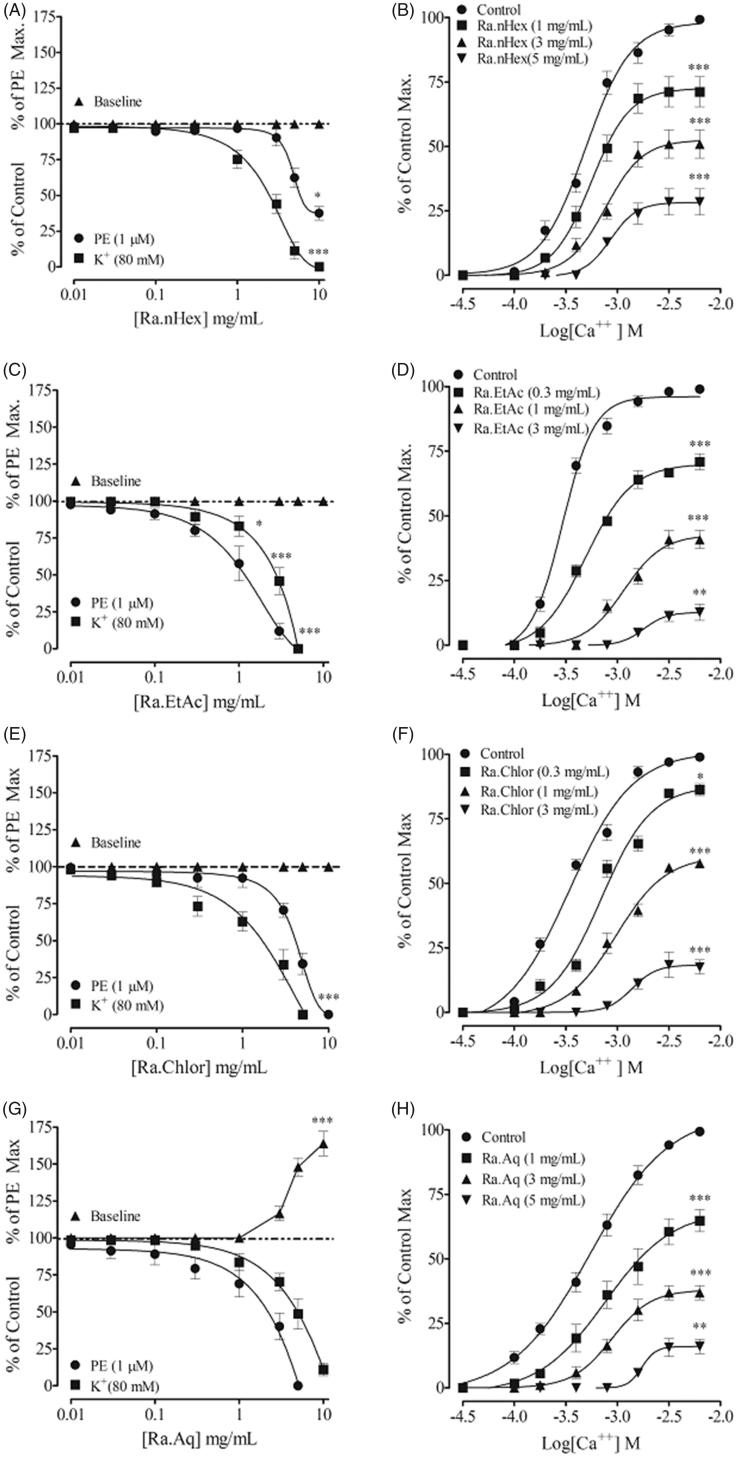
Concentration-dependent vasorelaxant response of all the fractions in rabbit aortic tissues and their respective sigmoidal CaCl_2_ curves constructed in Ca^2+^-free medium. Values shown are mean ± SEM. Five to six determinations.

Chloroform fraction exhibited similar effect to the parent extract, it induced relaxation of PE and high K^+^ pre-contractions with EC_50_ values of 5.14 (3.98–6.31) and 2.02 mg/mL (1.58–2.51), respectively (Figure 4(A)). Unlike the parent crude extract, the ethyl acetate fraction was found more potent against PE than high K^+^ pre-contractions with EC_50_ values of 3.01 (2.51–3.98) and 4.49 mg/mL (3.98–5.01), respectively (Figure 4(C)). The *n*-hexane fraction induced complete relaxation of high K^+^ pre-contractions, however, induced partial relaxation of PE pre-contractions with EC_50_ values of 3.01 (2.51–3.98) and 8.15 mg/mL (6.31–10), respectively (Figure 4(E)). The aqueous fraction also induced relaxation against PE and high K^+^ pre-contractions with EC_50_ value of 5.54 (3.21–7.44) and 7.69 mg/mL (5.38–10.0). Pre-treatment of the rabbit aortic rings with fractions caused a rightward shift in the CaCl_2_ sigmoidal CRCs, constructed in Ca^2+^ free medium, with suppression of maximum response (Figure 4(B,D,F,H)).

On base line tension, crude extract and fractions were without a stimulant effect (except the aqueous fraction), that caused a strong vasoconstrictor effect which is about 73% of the PE maximum (Figure 4(G)).

**Figure 5.**
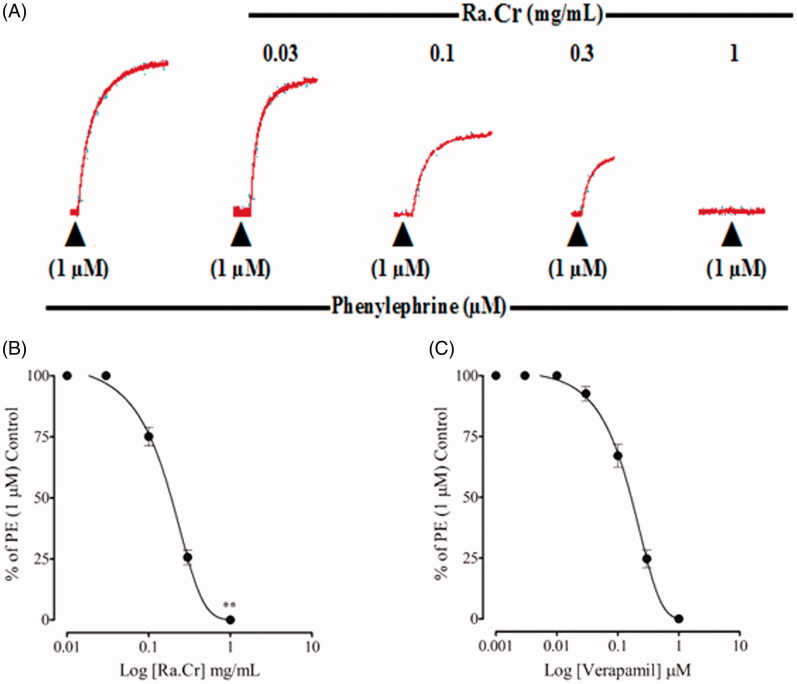
Typical tracing showing inhibitory effect of (A) increasing concentrations of the crude extract of *Rumex acetosa* (Ra.Cr) on the initial peak formation of phenylephrine (1 µM)-induced contractions in Ca^2+^-free medium. (B) The effect of Ra.Cr. (C) The effect of verapamil in isolated rabbit aorta preparations. Values shown are mean ± SEM. Five to six determinations.

In a series of experiments, an attempt was made to see possible effect of the crude extract on internal Ca^2+^ store(s), a transient contractile response was induced with PE (1 µM), in Ca^2+^ free/EGTA medium. Pre-treatment of the tissues with crude extract (0.03–1.0 mg/mL) suppressed, concentration dependently, the PE peak formation (Figure 5(B)), similar to that caused by verapamil (Figure 5(C)).

## Discussion

This investigation has provided for the first time detailed information on the antihypertensive potential of the extract and fractions of RA. We used two different rat models, normotensive and high salt-induced hypertensive. This protocol allowed us to observe effect of extract and fractions directly injected into the systemic circulation. We prefer this direct method because the tail cuff method is erratic where the absorption of extract or fractions into the systemic circulation is doubtful. Intravenous injection of the methanol extract of RA and its fractions caused a dose-dependent fall in MAP in both normotensive and hypertensive rats. However, the effect was more significant in the hypertensive rats, particularly at doses of 10 and 50 mg/kg. We also tested different fractions of the extract, all fractions were effective in lowering MAP in both models but like the parent extract they were more effective in the hypertensive rats. When compared to the parent crude extract, *n*-hexane and ethyl acetate fractions were similar, chloroform fraction was least potent and aqueous fraction was more potent.

Recently, Ahmad et al. (2016) has reported that the essential oil of *Rumex hastatus* possesses anticholinesterase activity. Anticholinesterase like substances is known to decrease BP and vascular resistance. To have insight into the antihypertensive effect of the extract and fractions *in vivo*, we tested the possibility if it might involve cholinergic pathway. Rats were pretreated with atropine, a muscarinic receptor antagonist (Arunlakhshana and Schild 1959). In the atropinized rats, the BP lowering effect of the crude extract and fractions was abolished at lower doses and partially ablated at higher doses. The effect of aqueous fraction on MAP was more sensitive to atropine pretreatment, indicating that muscarinic receptors activation is involved in the BP lowering effect of the extract and fractions.

Blood pressure is the product of peripheral vascular resistance and cardiac output (provide authentic reference). We investigated the effect of extract and fractions on vascular resistance using isolated vascular preparations *in vitro*. The vascular endothelium synthesizes and release number of important vasoactive compounds, which regulate vascular tone and BP. This includes NO, COX, endothelium-derived hyperpolarizing factor, endothelium-derived contracting factor and endothelin. These factors mediate the vascular effect of numbers of hormones including acetylcholine (Furchgott and Vanhoutte 1989). A previous study identified that ethanolic extract of RA accentuates NO production (Sun et al. 2015). We were interested to explore if additional vascular mechanisms are involved. In isolated rat thoracic aortic rings, extract and fractions induced endothelium-dependent vasorelaxation with relatively more potency in the aortic rings with intact endothelium than the denuded (except the aqueous fraction). We found that chloroform fraction was the most potent vasorelaxant. In the intact aortic rings pre-contracted with PE, the vasorelaxation induced by crude extract, ethyl acetate and chloroform fractions was attenuated in the presence of l-NAME, a nitric oxide synthase inhibitor (Fantel et al. 1997), indomethacin, a COX enzyme inhibitor (Moncada et al. 1978) and atropine. This pretreatment did not affect maximum relaxation, shifted the sigmoidal CRCs to the right. This indicates that the endothelium-dependent vasorelaxation is mediated by combination of NO, COX and muscarinic receptor-linked NO pathways. Data show that none of these mechanisms dominate. The relaxation induced by the *n*-hexane fraction was attenuated by l-NAME and atropine pretreatment, similar to the crude extract. Unlike the crude extract, this relaxation was abolished (>50%) by indomethacin pretreatment. Interestingly, the aqueous fraction induced endothelium-dependent vasoconstriction at lower concentrations followed by relaxation at higher concentrations. This vasoconstriction was abolished in the denuded aortic rings and intact rings pretreated with l-NAME, indomethacin and atropine. This can partly explain that the vasoconstriction induced by the aqueous fraction is endothelium dependent. We assume that the aqueous fraction releases endothelial-derived contractile factor(s) at lower concentration and relaxing factors at higher concentrations. The relaxing factors are sensitive to the inhibitory effect of indomethacin and atropine, as the vasorelaxation was abolished to >80% and >50%, respectively. This indicates that the crude extract contained combinations of vasoactive constituents; some are endothelium-derived relaxing like factors, which may equally distribute among the crude extract and ethyl acetate and chloroform fractions. However, the vasoconstrictor constituent(s) that were not prominent in the crude extract, shifted to the aqueous fraction. Furthermore, the atropine-sensitive (cholinergic type) constituents are shifted to the n-hexane and aqueous fractions, concentrated more in the later while the indomethacin-sensitive constituents are also concentrated in the aqueous fraction.

Failure of the relaxation induced by the crude extract and fractions in the presence of l-NAME, indomethacin and atropine or in denuded aortic rings indicates presence of constituents act through different vascular mechanism(s). We tested this hypothesis in rat aortic rings pre-contracted with high K^+^. High K^+^ induces vascular contraction through influx of Ca^2+^ into the cells via voltage-gated calcium channels (Bolton 1979). It is most probable that a substance which can inhibit high K^+^ induced contractions is considered a possible Ca^2+^ entry blocker (Godfraind et al. 1986). Interestingly, crude extract and fractions, with the exception of *n*-hexane, induced vasorelaxation against the high K^+^ pre-contractions. The aqueous fraction exhibited more potent response and chloroform showed the least. However, the *n*-hexane fraction induced partial (>50%) relaxation. Thus, the inhibition of high K^+^-induced contraction by the extract and fractions may reflect the effect on voltage-gated calcium channels and can possibly explain the endothelium-independent component of the vasodilator effect of the extract and fractions. The partial relaxation induced by the *n*-hexane fraction against high K^+^ pre-contractions, indicates involvement of additional mechanism(s), most probably activation of K^+^ channels because substances act through these channels become inactive in the presence of high K^+^.

There is sufficient evidence of heterogenicity of calcium channels (Koike et al. 1992), they are different in myocardium of rat and rabbit (Boyd et al. 1988) and brain of rat, frog and chicken (Suszkiw et al. 1987). We were interested to see if the extract and fractions of *R. acetosa* affect voltage-gated calcium channels differently in rabbit aorta. Rabbit aortic rings were pre-contracted with high K^+^ and PE where cumulative addition of the extract and fractions induced relaxation with varying potencies. The crude extract, *n*-hexane and chloroform fractions induced potent relaxation against high K^+^ than PE pre-contractions, similar to verapamil, a Ca^2+^ channel blocker (Godfraind et al. 1986) while the ethyl acetate and aqueous fractions induced potent relaxation against PE than high K^+^. This suggests that the extract and its fractions have inhibitory effect on Ca^2+^ entry either through voltage-gated calcium channels and/or ROCs. This hypothesis was further confirmed when pretreatment of the rabbit aortic rings with extract and fractions caused a rightward non-parallel shift in the CaCl_2_ sigmoidal CRCs, similar to verapamil. This indicates that Ca^2+^ entry blocking constituents are concentrated in the parent crude extract more compared to the fractions. The effect of the aqueous fraction against high K^+^ pre-contraction was interesting. It was >70 times potent as a vasorelaxant in the rat aortic rings compared to rabbit aortic rings. Unlike the rat aortic rings, aqueous fraction induced a vasoconstrictor effect at higher concentrations in the rabbit aortic rings. Similarly, the *n*-hexane fraction induced partial relaxation of high K^+^ pre-contraction in rat aortic rings, while it induced complete relaxation in the rabbit aortic rings. This discrepancy might be due to the presence of constituents act through diverse mechanisms or affect differently Ca^2+^ channels in same blood vessel from different species.

The nature of PE-induced contraction is biphasic, a fast (phasic phase) (Bohr 1963) and slow (tonic phase) phase (Yen et al. 1988). The fast component of the contraction is due to the mobilization of intracellular Ca^2+^ whereas the slow component (tonic phase) is directly dependent on the influx of Ca^2+^ (Scarborough and Carrier 1984), through receptor operated Ca^2+^ channels (Zhang et al. 2005).

Crude extract was tested in Ca^2+^ free/EGTA medium to see effect on Ca^2+^ release from the internal store using isolated rabbit aortic rings. When tested in Ca^2+^ free/EGTA medium, crude extract suppressed PE initial peak formation, similar to verapamil, suggesting its inhibitory effect on the release of Ca^2+^ from the internal store. These data indicate dual inhibitory effect of the extract of *R. acetosa* on Ca^2+^ movements; blocking effect on voltage-gated calcium channels and release of Ca^2+^ from the internal store.

## Conclusions

These data indicate that the extract of *R. acetosa* possesses an endothelium-dependent and independent vasodilator effect. The endothelium dependent vasodilator effect is mediated through NO of endothelium origin sensitive to l-NAME, indomethacin and atropine while the endothelium independent effect is mediated through dual inhibitory effect on Ca^+^ movement; entry via voltage-gated calcium channels and release from the internal Ca^2+^ store, which can possibly explain its antihypertensive effect in salt induced hypertensive and normotensive rats. The extract is seemed to be more effective in the hypertensive than normotensive rats, providing evidence to its medicinal importance in hypertension. The presence of a combination of vasoconstrictor constituents in the crude extract, separated into the aqueous fraction is meant to offset the excessive fall in BP usually associated with high doses of vasodilators. We could not identify the active constituents responsible for these activities. However, this study will have important impact on further research on this particular natural product.
